# CT-Imaging Based Analysis of Invasive Lung Adenocarcinoma Presenting as Ground Glass Nodules Using Peri- and Intra-nodular Radiomic Features

**DOI:** 10.3389/fonc.2020.00838

**Published:** 2020-05-27

**Authors:** Linyu Wu, Chen Gao, Ping Xiang, Sisi Zheng, Peipei Pang, Maosheng Xu

**Affiliations:** ^1^Department of Radiology, The First Affiliated Hospital of Zhejiang Chinese Medical University, Zhejiang Provincial Hospital of Traditional Chinese Medicine, Hangzhou, China; ^2^Department of Radiology, The First Clinical Medical College of Zhejiang Chinese Medical University, Hangzhou, China; ^3^Department of Pharmaceuticals Diagnosis, GE Healthcare, Hangzhou, China

**Keywords:** radiomics, tomography, X-ray computed, lung adenocarcinoma, ground-glass nodule, computational biology

## Abstract

**Objective:** To evaluate whether radiomic features extracted from intra and peri-nodular lesions can enhance the ability to differentiate between invasive adenocarcinoma (IA), minimally invasive adenocarcinoma (MIA), and adenocarcinoma *in situ* (AIS) manifesting as ground-glass nodule (GGN).

**Materials and Methods:** This retrospective study enrolled 120 patients with a total of 121 pathologically confirmed lung adenocarcinomas (85 IA and 36 AIS/MIA) from January 2015 to May 2019. The recruited patients were randomly divided into training (84 nodules) and validation sets (37 nodules), with a ratio of 7:3. The minority group in the training set was balanced by the synthetic minority over-sampling (SMOTE) method. The intra-, peri-nodular, and gross region of interests (ROI) were delineated with manual annotation. Image features were quantitatively extracted from each ROI on CT images. The minimum redundancy maximum relevance (mRMR) feature ranking method and the least absolute shrinkage and selection operator (LASSO) classifier were used to eliminate unnecessary features. The intra- and peri-nodular radiomic features were combined to produce the gross radiomic signature. A combined clinical-radiomic model was constructed by multivariable logistic regression analysis. The predicted performances of different models were evaluated using receiver operating curve (ROC) and calibration curve.

**Results:** The gross radiomic signature (AUC: training set = 0.896; validation set = 0.876) showed a good ability to discriminate the invasiveness of adenocarcinoma, comparing to intra-nodular (AUC: training set = 0.862; validation set = 0.852) or peri-nodular radiomic signature (AUC: training set = 0.825; validation set = 0.820). The AUC of the combined clinical-radiomic model was 0.917 for the training and 0.876 for the validation cohort, respectively.

**Conclusions:** The gross radiomic signature of intra- and peri-nodular regions improved the prediction ability and aided predicting the invasiveness of lung adenocarcinoma appearing as GGN.

## Introduction

Ground-glass nodule (GGN) on high-resolution computed tomography (HRCT) is defined as lesions showing hazy, increased attenuation that does not obscure underlying bronchial structures or pulmonary vessels (1–3). Early-stage lung adenocarcinoma nodules often manifest GGN associated with a pathological, lepidic growth pattern (4). According to the pathology classification in 2011, lung adenocarcinoma has been divided into pre-invasive lesions including atypical adenomatous hyperplasia (AAH) and adenocarcinoma *in situ* (AIS), minimally invasive adenocarcinoma (MIA), and invasive adenocarcinoma (IA) (5–7). The suggested therapeutic strategy for each differs according to different subgroups of adenocarcinoma classification. Compared with IA, AIS, and MIA can be treated with wedge or segmental resection with a 100% or near 100% of 5-year survival rate (8). Therefore, preoperative, non-invasive radiological assessment of the invasiveness of lung adenocarcinoma is essential.

Histology is currently the gold standard to assess the invasiveness of lung adenocarcinoma. However, it is generally difficult to assess the overall invasiveness of GGNs before surgery. Traditional evaluation using CT can determine values such as tumor size, CT attenuation value, and the percentage of the solid component in GGN, which can help differentiate the invasiveness of the adenocarcinoma (4, 6, 7). Yet, results are subjective and depend widely on the experience of those reading the images. From texture analysis to radiomic studies (9–11), these radiomic features inside the tumor help to differentiate the histological invasiveness of lung adenocarcinoma appearing as GGN. Luo et al. (10) constructed a nomogram model combining the qualitative CT imaging features and the radiomic features extracted from intratumor. This showed an AUC of 0.903. The radiomic features and mean CT value of GGN ≤ 10 mm was combined. The constructed nomogram model was designed to achieve a C-index of 0.707–0.721 (11). However, these studies (9–11) only focused on the radiomic features inside the tumor and ignored the regions surrounding the tumor which may be helpful for differentiation.

Little work was reported on studying GGNs using peri-nodular radiomic feature for prediction the invasiveness of lung cancer. Recent studies indicate that the tumor microenvironment and the associated abnormalities play an important role in tumorigenesis, including lung cancer (12, 13). Therefore, we hypothesized that analyzing the peritumoral region would provide valuable insight for the intra-nodular radiomic analysis. Our aim was to evaluate whether radiomic features extracted from intra- and peri-nodular regions of lung nodules on CT images can improve the ability to determine the histological invasiveness of adenocarcinoma appearing as GGNs.

## Materials and Methods

The institutional review board in our hospital approved this retrospective study and informed consent was waived.

### Patients

From January 2015 to May 2019, 382 consecutive patients with 389 pulmonary adenocarcinomas confirmed by operative pathology were reviewed based on the 2015 the International Association for the Study of Lung Cancer (IASLC), the American Thoracic Society (ATS), and the European Respiratory Society (ERS) classification of lung adenocarcinoma in our institution. The inclusion criteria in the study were as follows:(1) the presence of a chest CT scan with a thin-slice thickness (0.75 mm) before surgical treatment within one month; (2) lung adenocarcinomas presenting as GGN (including pure GGN and part-solid nodule) with a diameter ≤ 30 mm with lung window [level: −600 Hounsfield unit (HU); width: 1300 HU]; (3) no chemotherapy, radiotherapy before surgery; (4) no other malignant tumor history and distant metastasis. The exclusion criteria were as following: (1) biopsy, radiotherapy, chemotherapy or surgical resection of lung malignant tumor performed before CT examination; (2) thin-slice images with low dose scan, different slice thickness, or different reconstruction algorithm; (3) multiple GGNs in the same pulmonary lobe. A total of 121 GGNs from 120 patients matched the search criteria research.

### CT Image Qualitative Evaluation

Blinded to each patient's information, two thoracic radiologists (with 10 and 5 years of experience in chest CT) evaluated all the thin-slice CT images and the decision was reached by consensus. The following clinical parameters, including gender, age, the maximal diameter of the nodule, nodular type, position, and morphology characteristics were derived and recorded. The morphology characteristics included spiculation, lobulation, pleural indentation sign, air bronchogram, vacuole, and vessel convergence sign.

### Image Acquisition and Segmentation

All primary and intra-cross validation cohorts underwent scanning at our institution with a Somatom Sensation 64 (Siemens Healthcare, Germany). CT scan parameters were as follows: detector collimation = 0.6 mm × 64; pitch = 1.4; tube voltage = 120 kV; automatic tube current modulation; reconstructed section thickness and interval = 0.75 and 0.5 mm; field of view = 300 mm; matrix = 512 × 512;reconstructed convolution function = B31f.

An open software (ITK-SNAP 3.6.0 available at www.itksnap.org) was used to manually segment the thin-slice CT images with DICOM format. The image segmentation of GGNs was contoured along the boundary of each nodule on all axial images in the lung window setting (window level, −600 HU; window width, 1,300 HU) on a layer by layer basis until the entire lung nodule was covered.

The reproducibility of inter-observer and intra-observer segmentation was initially assessed with 30 randomly chosen GGNs by thoracic radiologists with 10 years' experience (reader A, PX) and 5 years' experience (reader B, LW) independently. The two radiologists were blinded to each other's segmentations. Then reader B(LW) repeated the same procedure after 1 month. Inter- and intra-class correlation coefficients (ICCs) was used to assess the intra- and inter-observer agreement of feature extraction. It indicated a good agreement if the ICC > 0.75, and reader B(LW) performed the remaining image segmentation.

**Figure 1:**
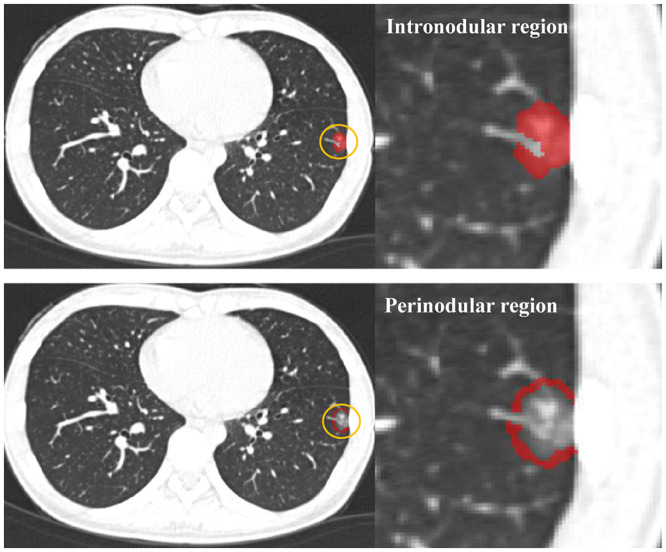
The intra-nodular region and the peri-nodular region (the ring 2 mm region) were drawn on an axial image.

The peri-nodular region was dilated 2 mm in three dimensions automatically using AK software (Analysis Kit, GE Healthcare, US) after the intra-nodular region was drawn. All bronchi, large vessels, vacuole, and normal tissue beyond the pleura were manually excluded from each region of interest (ROI). Figure 1 showed the peri-nodular region with a circle of the dilated 2 mm region lung parenchyma around the nodule.

### Feature Selection and Radiomic Signature Building

The segmented intra- and peri-nodular region was imported into the AK Software (Analysis Kit, GE Healthcare, US) for ROI texture feature extraction. Three hundred and ninety-six radiomic features including the histogram, form-factor, gray-level co-occurrence matrix (GLCM), and run-length matrix (RLM) were calculated. Before selecting features, the feature value of all patients was normalized with a *Z*-score [(*x* – μ)/σ], (where *x* is the feature value and μ represents the average of the feature values among all patients, and σ stands for the corresponding standard deviation) to remove the unit limit for each feature before applying it to the machine learning model for classification. Subsequently, all patients were divided into training cohorts and validation cohorts according to 7:3 randomly.

Due to sample imbalance (the number of MIA/AIS is much less than that of IA), the synthetic minority over-sampling (SMOTE) algorithm (14) was used to balance the minority group in the training set. First, the data of the training set was equalized via the SMOTE, so that the two types of training samples were close to 1:1. Then the feature dimension reduction and machine learning modeling were based on the equalized data. After the model was established, all predictions were based on the real training set and validation set. The SMOTE algorithm was used to reduce the impact of sample imbalance on the model.

Two feature selection methods, the minimum redundancy maximum relevance (mRMR) and the least absolute shrinkage and selection operator (LASSO) were used to select the most useful predictive features in the training cohort. Firstly, by using a multivariate ranking method mRMR method, the features were ranked according to their relevance-redundancy index based on the heuristic scoring criteria, the top 20 features with high-relevance were selected. Then, a LASSO classifier was conducted using 10-fold cross-validation on the training cohort to choose the optimized subset of features and build a radiomic signature. The corresponding coefficients were evaluated. The radiomic signature (rad-score) was calculated by summing the selected texture features that were weighted by their respective coefficients. All rad-scores between the IA and AIS/MIA group were compared on the training set and validation set, respectively.

### Development and Validation of the Intra-Nodular Radiomic, the Peri-nodular Radiomic, the Gross Radiomic, and Construction of the Radiomic Model

Before performing the multivariate logistic regression analysis, collinearity diagnosis was performed using correlation matrix heat map. Radiomic signatures of intra- and peri-nodular features were built. Furthermore, using the same method as described above, the intra- and peri-nodular radiomic features were combined to produce additional intra- and peri-nodular radiomic features (the gross radiomic signature). The area under the receiver operator characteristic (ROC) curve (AUC) was used to assess the performance of the different models. The radiomic signature with the highest AUC was selected.

For clinical variables and visual evaluation parameters on CT, the significance of associations with the invasiveness of adenocarcinoma was evaluated using the univariate logistic analysis. Variables with *P* ≥ 0.05 in univariable analysis were eliminated. Finally, significant clinical risk factors and the radiomic signature with the highest AUC were introduced into the step-wise multivariate logistic regression analysis to build the clinical-radiomic model using the likelihood ratio test with Akaike's information criterion (AIC) as the stopping rule (15). The sensitivity, specificity, accuracy, positive-predictive value (PPV), and negative-predictive value (NPV) for the models in both the primary and the validation cohort were calculated based on the Youden index (16).

A calibration curve was used to investigate the performance of the clinical-radiomic model. The Hosmer–Lemeshow test was performed to evaluate the degree of fit of the clinical-radiomic model. The predictive performance of the clinical model, the gross rad-score model, and the clinical-radiomic model was quantified by AUC based on ROC curve analysis.

### Statistical Analysis

The LASSO logistic regression was conducted by 10-fold cross-validation based on minimum criteria. The normality test was first conducted for the continuous variables. If the data followed a normal distribution, the independent sample *t*-tests were used for the normally distributed continuous variables. The Mann–Whitney *U*-test was used for the non-normally distributed continuous variables. A Chi-square test was used to compare the differences for categorical variables of clinical data between the two groups. A backward stepwise selection was applied using the likelihood ratio test with Akaike's information criterion as the stopping rule. Differences between various AUCs was evaluated using DeLong's method (17). Statistical analysis was conducted with R software (version 3.3.3; https://www.r-project.org). The reported statistical significance levels were all two-sided, with statistical significance set at 0.05. The “stats” package (http://www.personal.psu.edu/drh20/R/html/stats/html/stats-package.html) was used for multiple logistic regression analysis. ROC curve analysis was performed using the “pROC” and “ROC.TEST” software packages for R.

## Results

### Patient Characteristics

**Figure 2:**
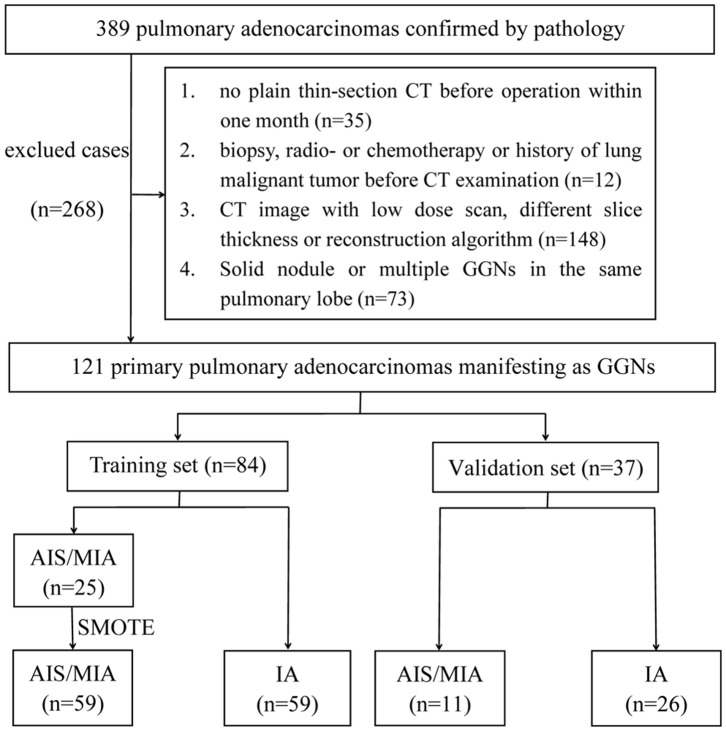
Flowchart of study population.

A total of 120 consecutive patients [34 men, 86 women; mean age = 55.9 years ± 11.9 (standard deviation); range = 26–81 years] with 121 primary pulmonary adenocarcinomas manifesting as GGNs were enrolled from January 2015 to December 2017. In this study, 268 lung adenocarcinomas were excluded. Of the 121 GGNs, 85 were invasive adenocarcinomas, 27 were microinvasive adenocarcinomas, and 9 were *in situ* adenocarcinomas. The specific flow chart was presented in Figure 2.

There were no statistical differences between the training set and the validation set in patients' gender, age, and CT imaging features (Table S1). Characteristics of qualitative CT imaging features and demographic features between AIS/MIA and IA both in the training and testing set of our study were presented in Table 1.

**Table 1:** Characteristics of the training and validation cohorts.

**Characteristics**	**Training set (*****n*** **=** **84)**			**Validation set (*****n*** **=** **37)**		
	**AIS/MIA (*n* = 25)**	**IA (*n* = 59)**	***P***	**AIS/MIA (*n* = 11)**	**IA (*n* = 26)**	***P***
**Gender**						
Female	16 (64.00%)	43 (72.88%)	0.416	9 (81.82%)	19 (73.08%)	0.695
Male	9 (36.00%)	16 (27.12%)		2 (18.18%)	7 (26.92%)	
Age (year)	52.44 ± 12.71	60.22 ± 10.24	0.004	52.73 ± 11.23	56.96 ± 10.63	0.283
**Location**			0.546			0.289
Right upper lobe	13 (52.00%)	22 (37.29%)		7 (63.64%)	9 (34.62%)	
Right middle lobe	1 (4.00%)	6 (10.17%)		0 (0.00%)	4 (15.38%)	
Right lower lobe	3 (12.00%)	7 (11.86%)		3 (27.27%)	4 (15.38%)	
Left upper lobe	6 (24.00%)	12 (20.34%)		1 (9.09%)	6 (23.08%)	
Left lower lobe	2 (8.00%)	12 (20.34%)		0 (0.00%)	3 (11.54%)	
**Spiculation**			0.001			0.014
Absent	20 (80.00%)	23 (38.98%)		10 (90.91%)	12 (46.15%)	
Present	5 (20.00%)	36 (61.02%)		1 (9.09%)	14 (53.85%)	
**Lobulation**			0.003			0.719
Absent	16 (64.00%)	17 (28.81%)		6 (54.55%)	11 (42.31%)	
Present	9 (36.00%)	42 (71.19%)		5 (45.45%)	15 (57.69%)	
**Pleural Indentation**			0.129			0.141
Absent	18 (72.00%)	32 (54.24%)		9 (81.82%)	13 (50.00%)	
Present	7 (28.00%)	27 (45.76%)		2 (18.18%)	13 (50.00%)	
**Air Bronchogram**			0.651			0.015
Absent	19 (76.00%)	42 (71.19%)		11 (100.00%)	15 (57.69%)	
Present	6 (24.00%)	17 (28.81%)		0 (0.00%)	11 (42.31%)	
**Vacuole**			0.923			0.083
Absent	22 (88.00%)	54 (91.53%)		9 (81.82%)	26 (100.00%)	
Present	3 (12.00%)	5 (8.47%)		2 (18.18%)	0 (0.00%)	
**Vessel Convergence**			0.034			0.091
Absent	9 (36.00%)	9 (15.25%)		5 (45.45%)	4 (15.38%)	
Present	16 (64.00%)	50 (84.75%)		6 (54.55%)	22 (84.62%)	
**Nodule Type**			0.309			0.699
Pure GGN	10 (40.00%)	11 (18.64%)		4 (36.36%)	7 (26.92%)	
Part-solid GGN	15 (60.00%)	48 (81.36%)		7 (63.64%)	19 (73.08%)	
**Diameter**	11.72 ± 6.13	14.81 ± 5.86	0.032	9.36 ± 4.11	14.62 ± 4.88	0.004

*AIS, adenocarcinoma in situ; MIA, minimally invasive adenocarcinoma; IA, invasive adenocarcinoma; GGN, ground-glass nodule*.

### Interobserver and Intraobserver Reproducibility of Radiomic Feature Extraction

The intra-reader ICC between two measurements by reader A ranged from 0.821 to 0.932. The inter-reader ICC between reader A and reader B ranged from 0.789 to 0.868. The results indicated a favorable inter- and intra-observer reproducibility for feature extraction.

### The Radiomic Signature Building and Diagnostic Validation

**Figure 3:**
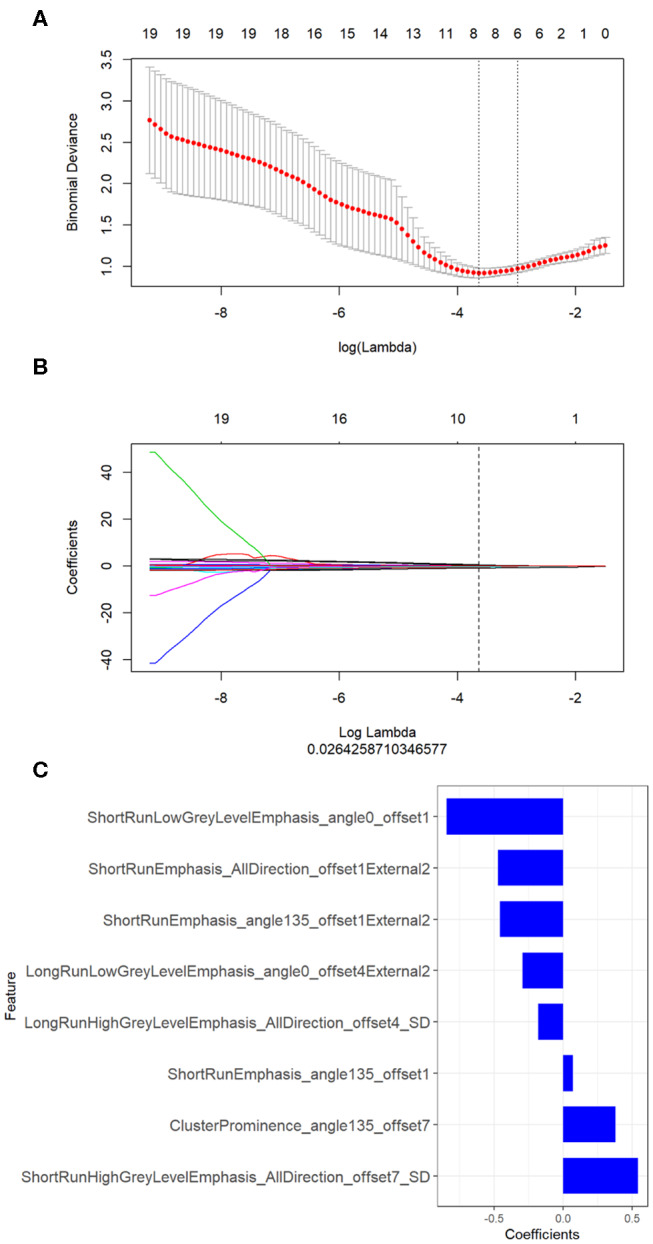
Texture feature selection by using the least absolute shrinkage and selection operator (LASSO) about the gross radiomics. **(A)** Optimal feature selection according to AUC value; **(B)** LASSO coefficient profiles of the 20 radiomic features. A vertical line was drawn at the selected value using 10-fold cross-validation, where the optimal λ resulted in eight non-zero coefficients; **(C)** The selected radiomic features and their coefficients about the gross radiomic signature.

Three hundred and ninety-six texture features were extracted from AK software within the intra- and peri-nodular region. Then the mRMR was firstly performed to eliminate the redundant and irrelevant features, 20 features were retained. Texture feature selection was conducted using the LASSO about the intra- and peri-nodular regions, along with the gross signatures (Figures 3A,B; Figures S1A–D).

The LASSO classifier provided the optimal radiomic signature to construct the final model with values of AUC = 0.862, 0.825, and 0.896 for intra-, peri-nodular, and gross signatures in the training cohort, respectively (Table 2). The ROC curve of the radscore with intra- and peri-nodular were showed in Figure S2 both in training and validation set. For each group, the gross signature that combined the peri- and intra-nodular features with the highest AUC was selected.

**Table 2:** Predictive value between five different models in the training and validation cohort.

**Signature**	**AUC**	**95%CI**	**Sensitivity**	**Specificity**	**Accuracy**	**PPV**	**NPV**
**Training cohort**							
Intra-nodular rad-score	0.862	0.778–0.946	0.654	0.933	0.848	0.810	0.862
Peri-nodular rad-score	0.825	0.735–0.915	0.923	0.583	0.686	0.490	0.946
Gross rad-score	0.896	0.826–0.967	0.808	0.867	0.849	0.724	0.912
Clinical features	0.718	0.593–0.842	0.840	0.500	0.698	0.700	0.692
Clinical-radiomics	0.917	0.860–0.974	0.979	0.658	0.837	0.783	0.962
**Validation cohort**							
Intra-nodular rad-score	0.852	0.718–0.986	0.900	0.800	0.829	0.643	0.952
Peri-nodular rad-score	0.820	0.679–0.961	0.800	0.760	0.771	0.571	0.905
Gross rad-score	0.876	0.747–1.000	0.800	0.920	0.886	0.800	0.920
Clinical features	0.768	0.570–0.966	0.900	0.533	0.743	0.720	0.800
Clinical-radiomics	0.876	0.739–1.000	0.952	0.643	0.829	0.800	0.900

*AUC, area under the receiver operator characteristic curve; 95%CI, 95% confidence interval; PPV, positive predictive value; NPV, negative predictive value*.

**Figure 4:**
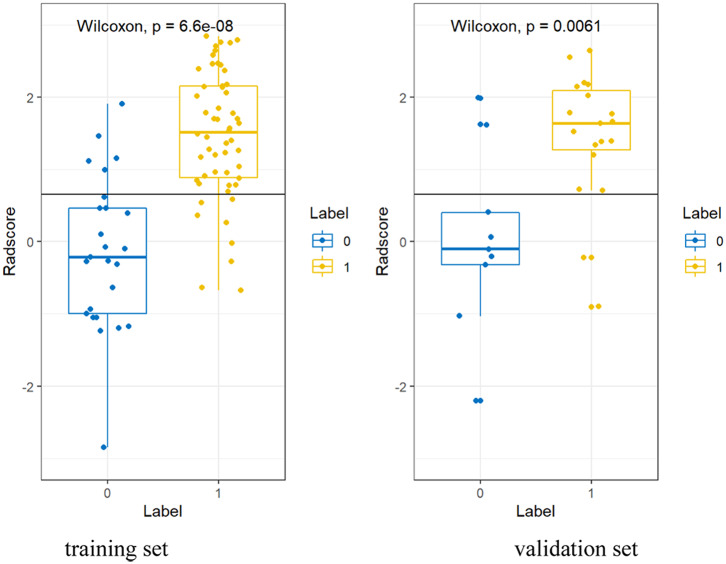
The boxplot about gross rad-scores between AIS/MIA and IA both in the training and validation sets.

Based on the formula of the radiomic signature, the three rad-scores were calculated. The calculation formula for intra-, peri-nodular, and gross signatures were presented in the Supplementary Material. The waterfall plot showed the rad-score for the patient vividly (Figure 3C, Figures S1E,F). All the rad-scores of IA were significantly higher compared to the AIS/MIA in both the training set and validation set, and results were represented by boxplot in Figure 4, Figures S1G,H. The intra-nodular rad-score, peri-nodular rad-score, and gross rad-score demonstrated good predictive efficacy in the training and validation cohorts (intra-nodular rad-score, the AUC values were 0.862 and 0.852, respectively; peri-nodular rad-score, the AUC values were 0.825 and 0.820, respectively; gross rad-score, the AUC values were 0.896 and 0.876, respectively). The relevant results were shown in Table 2.

**Figure 5:**
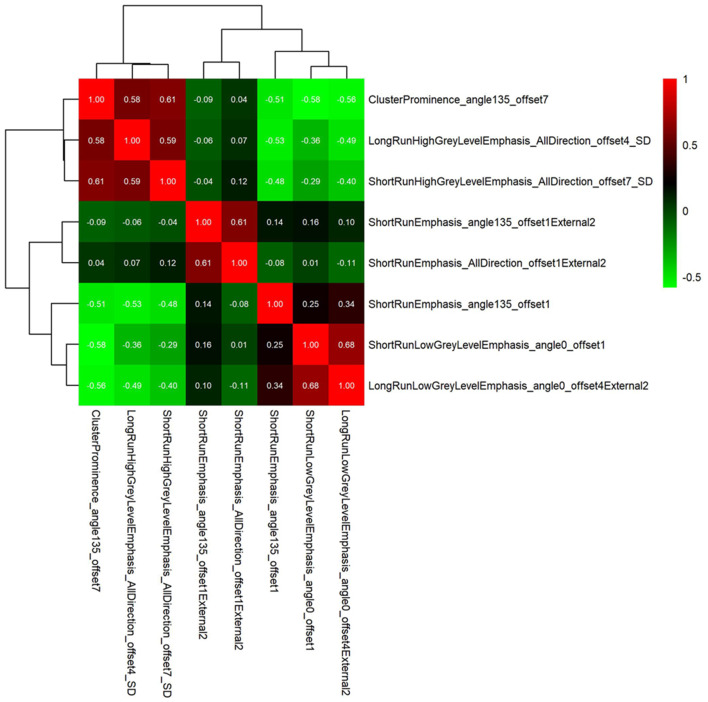
The correlation matrix heat map showing no collinearity between the gross features.

The correlation coefficients between the intra-, peri-nodular, and gross signatures were all <0.7 using correlation matrix heat map, which indicated no collinearity between features (Figure 5, Figure S3).

### Development and Predictive Performance of the Clinical-Radiomic Model

Univariate logistic regression analysis showed that age, the maximum diameter of the nodule, spiculation, lobulation, pleural indentation, and vessel convergence were risk factors of invasive adenocarcinoma. After multivariate analysis, the gross rad-score (OR: 14.420, 95%CI: 3.700–56.180; *p* < 0.001) and the maximal diameter of nodule (OR: 0.800, 95%CI: 0.605–0.980; *p* < 0.05) with the lowest AIC value (AIC = 60.64) was identified as the best model (Table 3). The average diameter of IA was larger than that of AIS or MIA both in the training set (14.81 ± 5.86 vs. 11.72 ± 6.13 mm; *p* < 0.05) and the validation set (14.62 ± 4.88 vs. 9.36 ± 4.11; *p* < 0.01).

**Table 3:** Risk factors for invasiveness of the ground-glass nodule (GGN) in the training set.

	**Univariate logistic regression**		**Multivariate logistic regression**	
**Characteristics**	**OR (95%CI)**	***p***	**OR (95%CI)**	***p***
**Intra-nodular model**				
Rad-score	5.027 (2.500–10.105)	<0.001	NA	NA
**Peri-nodular model**				
Rad-score	7.148 (2.722–18.771)	<0.001	NA	NA
**Gross model**				
Rad-score	5.435 (2.548–11.429)	<0.001	14.420 (3.700–56.180)	<0.001
**Clinical model**				
Age	1.056 (1.011–1.104)	0.015	NA	NA
Diameter	1.149 (1.037–1.273)	0.008	0.800 (0.605–0.980)	0.031
Spiculation	4.800 (1.599–14.410)	0.005	NA	NA
Lobulation	3.200 (1.231–8.317)	0.016	NA	NA
Vessel convergence	3.542 (1.225–10.236)	0.019	NA	NA

*CI, confidence interval; GGN, ground-glass nodule; OR, odds ratio; NA, not available*.

**Figure 6:**
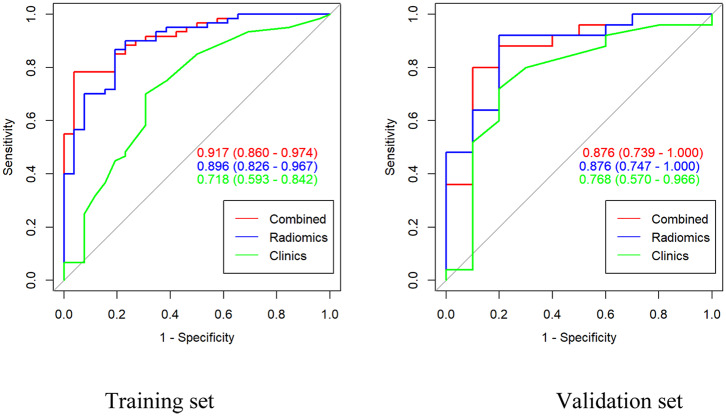
Area under the curve (AUC) of the gross signatures, clinical model, and the combined model in the training cohort and the validation cohort.

The ROC curves for the gross radiomic model, clinical model, and combined clinical-radiomic model on the training cohort and validation cohort were shown in Figure 6. In the training cohort, the clinical-radiomic model combining the gross signature with the clinical parameter (the maximal diameter of nodule) showed best predictive performance (AUC = 0.917, 95%CI: 0.860–0.974) with good sensitivity (0.979), specificity (0.658), and accuracy (0.837) among the intra-nodular rad-score, the peri-nodular rad-score, the gross rad-score, and the clinical features (Table 2). In the validation cohort, the clinical-radiomic model also showed a good performance (AUC = 0.876, 95%CI: 0.739–1.000) with high sensitivity (0.952), specificity (0.643), and accuracy (0.829) (Table 2). However, the AUC values of clinical parameters in differentiating AIS/MIA from IA were only 0.718 (95%CI: 0.593–0.842) and 0.768 (95% CI: 0.570–0.966) in the training and validation set, respectively (Table 2). The corresponding accuracy, sensitivity, specificity values, PPV, and NPV of all the models were calculated and presented in Table 2.

The calibration curve of the gross signature in the training and validation set is presented in Figure S4. There was no statistical significance both in the training and validation cohort (*P* = 0.275 and 0.197, respectively) by the Hosmer–Lemeshow test, which suggested that the combined model predictions and actual results were in good agreement. Statistically significant differences were observed in the training cohort (Gross model vs. Clinical model: 0.896 vs. 0.717, *p* < 0.001; Combined model vs. Clinical model: 0.917 vs. 0.717, *p* < 0.001). However, there was no difference between the gross model and clinical model in the training cohort (0.917 vs. 0.896, *P* = 0.334).

## Discussion

In this study, we evaluated the ability of imaging features from intra-, peri-nodular, and gross regions which combined the intra- and peri-nodular radiomic features to discriminate AIS/MIA from IA. It was found that the gross radiomic signature improved the discrimination ability better than the intra- and peri-nodular radiomic signatures alone. From this finding, a clinical radiomic model combining the gross radiomic signature and the maximum diameter of the nodule was also created and validated. The clinical-radiomic model and the gross radiomic model outperformed the intra-, peri-nodular signature, and the clinical parameter-based models.

We found that the radiomic features of the Cluster Prominence and the gray level run-length matrix (GLRLM) extracted from the tumor and peritumor had the ability to differentiate the invasiveness of adenocarcinoma. In our study, all the Cluster Prominence and the GLRLM textural features of IA showed significantly higher levels than that of AIS/MIA. This suggest that IA demonstrated more heterogeneity than the MIA/AIS both in the tumor and the surrounding area. Specifically, heterogeneity represented by Cluster Prominence and GLRLM features were consistent with the medical literature (18–20). All these parameters described the image characteristics in the form of heterogeneity or asymmetry. In medical imaging, high values of cluster prominence represent a larger peak for the image gray level value. Typically, the gray level difference between the forms is large. Therefore, GLCM can represent the heterogeneity of local tissue as it analyzes texture changes through the relationship between neighboring pixels. Several studies have reported that the GLRLM texture analysis can assess tumor heterogeneity (19, 20). Karacavus et al. (20) founded that GLRLM textural feature in the tumor approach could be useful in the discrimination of tumor stage. Our study regarding the radiomic signature also represented the high heterogeneity of in the tumor.

In contrast, most prior radiomic approaches to discriminate the invasiveness of adenocarcinoma have been focused solely within the extent of the tumor itself (21–25). Fan et al. (22) found that the radiomic feature from nodules was able to differentiate invasiveness of adenocarcinomas with high sensitivity, specificity, and accuracy in the primary and validation sets. However, the tumor microenvironment has not been relatively explored and the radiomic signature extracted from peripheral lung parenchyma maybe enable enhanced tumor invasiveness prediction. Radiomic features extracted from the tumor and peritumor can provide information on both the tumor and its microenvironment, which play an important role on the prediction of lymph node metastasis, post-surgical recurrence risk, discrimination of adenocarcinomas from granulomas (26–28). Our study suggested that the GLRLM features of the surrounding area of tumor in IA was also higher than that in MIA/AIS, which indicated that the periphery of IA was also more heterogeneous. Specifically, the radiomic signature extracted from peripheral lung parenchyma may be a reliable indicator of the state of the tumor microenvironment, which may differ across different degrees of tumor invasiveness. The peri-nodular region was defined as the surrounding 2 mm encompassing the nodule in our study. This distance of surrounding area was set after a previous study conducted by Sun et al. (29).

Furthermore, the clinical-radiomic model that combined the maximum diameter of the nodule and the gross radiomic signatures was built and analyzed in our study. The clinical-radiomic signature demonstrated excellent discrimination both in the training and validation cohorts (with respective AUCs of 0.917, 0.876). The mean maximal diameter of IA was larger than that of MIA/AIS. This finding agrees with the suggestions of Lee et al. (24) who found that the maximal diameter of invasive adenocarcinoma was higher than that of pre-invasive adenocarcinoma. However, the clinical-radiomic model was no better than the gross radiomic model in the validation cohort. This finding may be due to the fact that there was only one clinical feature and the gross radiomic features played an overwhelming weight in the clinical-radiomic model.

However, there are several limitations to our study. Firstly, benign lesions, including atypical adenomatous hyperplasia, were not included in our research. Secondly, there was a relatively limited sample size because this was a retrospective study and inclusion criteria was strict. In our research, all the patients underwent CT scanning at our institution on the same Somatom Sensation 64 (Siemens Healthcare, Germany) with standardization in scanning protocols, post-processing. Therefore, further external validation is expected to validate the identifying power of the models in a multicenter hospital with a larger cohort. Third, it still was a challenge to segment nodules by an automatic technique and all the ROI in the study were manually segmented with much time and energy. Finally, confounding effects by small blood vessels or bronchi may not be completely removed as the ROIs were manually drawn.

In conclusion, while some limitations are present, the current study provides strong evidence to suggest that the gross radiomic signature comprised of the peri- and intra-modular signatures offer a powerful diagnostic tool when combined with the clinical-radiomic model for discriminating the invasiveness of lung adenocarcinoma appearing as GGN. While histology may remain the gold standard, the method proposed herein provides a convincing, non-invasive method for initial diagnosis before the surgery.

## Data Availability Statement

The datasets generated for this study are available on request to the corresponding author.

## Ethics Statement

The studies involving human participants were reviewed and approved by The Ethics Committee of the First Affiliated Hospital of Zhejiang Chinese Medical University. Written informed consent for participation was not required for this study in accordance with the national legislation and the institutional requirements.

## Author Contributions

MX and LW: conception, design, writing-review, and editing. LW, CG, and PX: data curation and processing. LW, CG, PX, and SZ: formal analysis. MX and PX: funding acquisition. LW and PP: methodology. MX: project administration and supervision. LW: writing-original draft.

## Conflict of Interest

PP was employed by company GE Healthcare, Hangzhou, China. The remaining authors declare that the research was conducted in the absence of any commercial or financial relationships that could be construed as a potential conflict of interest.
