# Supplementary material for: CT-Imaging Based Analysis of Invasive Lung Adenocarcinoma Presenting as Ground Glass Nodules Using Peri- and Intra-nodular Radiomic Features
Source: Front Oncol. 2020 May 27;10:838. doi: 10.3389/fonc.2020.00838 (PMC7267037; doi:10.3389/fonc.2020.00838)
Supplement: Supplementary file 1 [file Data_Sheet_1.docx]

***Supplementary material***

**1．Supplementary Table**

| **Characteristics** | **Size** | **Training Set (n=84)** | **Validation Set**  **(n=37)** | **Statistics** | ***P*** |
| --- | --- | --- | --- | --- | --- |
| **Gender** |  |  |  |  |  |
| Female | 87 | 59(70.24%) | 28(75.68%) | 0.376 | 0.540 |
| Male | 34 | 25(29.76%) | 9(24.32%) |  |  |
| **Age(year)** | 121 | 57.90±11.52 | 55.70±10.83 | -0.986 | 0.326 |
| **Position** |  |  |  |  |  |
| Right upper lobe | 51 | 35(41.67%) | 16(43.24%) | 2.505 | 0.644 |
| Right middle lobe | 11 | 7(8.33%) | 4(10.81%) |  |  |
| Right lower lobe | 17 | 10(11.90%) | 7(18.92%) |  |  |
| Left upper lobe | 25 | 18(21.43%) | 7(18.92%) |  |  |
| Left lower lobe | 17 | 14(16.67%) | 3(8.11%) |  |  |
| **Spiculation** |  |  |  |  |  |
| Absent | 65 | 43(51.19%) | 22(59.46%) | 0.706 | 0.401 |
| Present | 56 | 41(48.81%) | 15(40.54%) |  |  |
| **Lobulation** |  |  |  |  |  |
| Absent | 50 | 33(39.29%) | 17(45.95%) | 0.470 | 0.493 |
| Present  **Pleural Indentation** | 71 | 51(60.71%) | 20(54.05%) |  |  |
| Absent | 72 | 50(59.52%) | 22(59.46%) | 0.000 | 0.995 |
| Present | 49 | 34(40.48%) | 15(40.54%) |  |  |
| **Air Bronchogram** |  |  |  |  |  |
| Absent | 87 | 61(72.62%) | 26(70.27%) | 0.070 | 0.791 |
| Present | 34 | 23(27.38%) | 11(29.73%) |  |  |
| **Vacuole** |  |  |  |  |  |
| Absent | 111 | 76(90.48%) | 35(94.59%) | 0.160 | 0.689 |
| Present | 10 | 8(9.52%) | 2(5.41%) |  |  |
| **Vessel Convergence** |  |  |  |  |  |
| Absent | 27 | 18(21.43%) | 9(24.32%) | 0.124 | 0.724 |
| Present | 94 | 66(78.57%) | 28(75.68%) |  |  |
| **Nodule Type** |  |  |  |  |  |
| Pure GGN | 32 | 21(25.00%) | 11(29.73%) | 0.295 | 0.587 |
| Part-solid GGN | 89 | 63(75.00%) | 26(70.27%) |  |  |
| **Diameter** | 121 | 13.89±6.07 | 13.05±5.21 | -0.730 | 0.467 |

Table S1. The comparison of the training set with the validation set in patients’ gender, age, and CT imaging features

**2. Supplementary Figures**

(B)

(A)


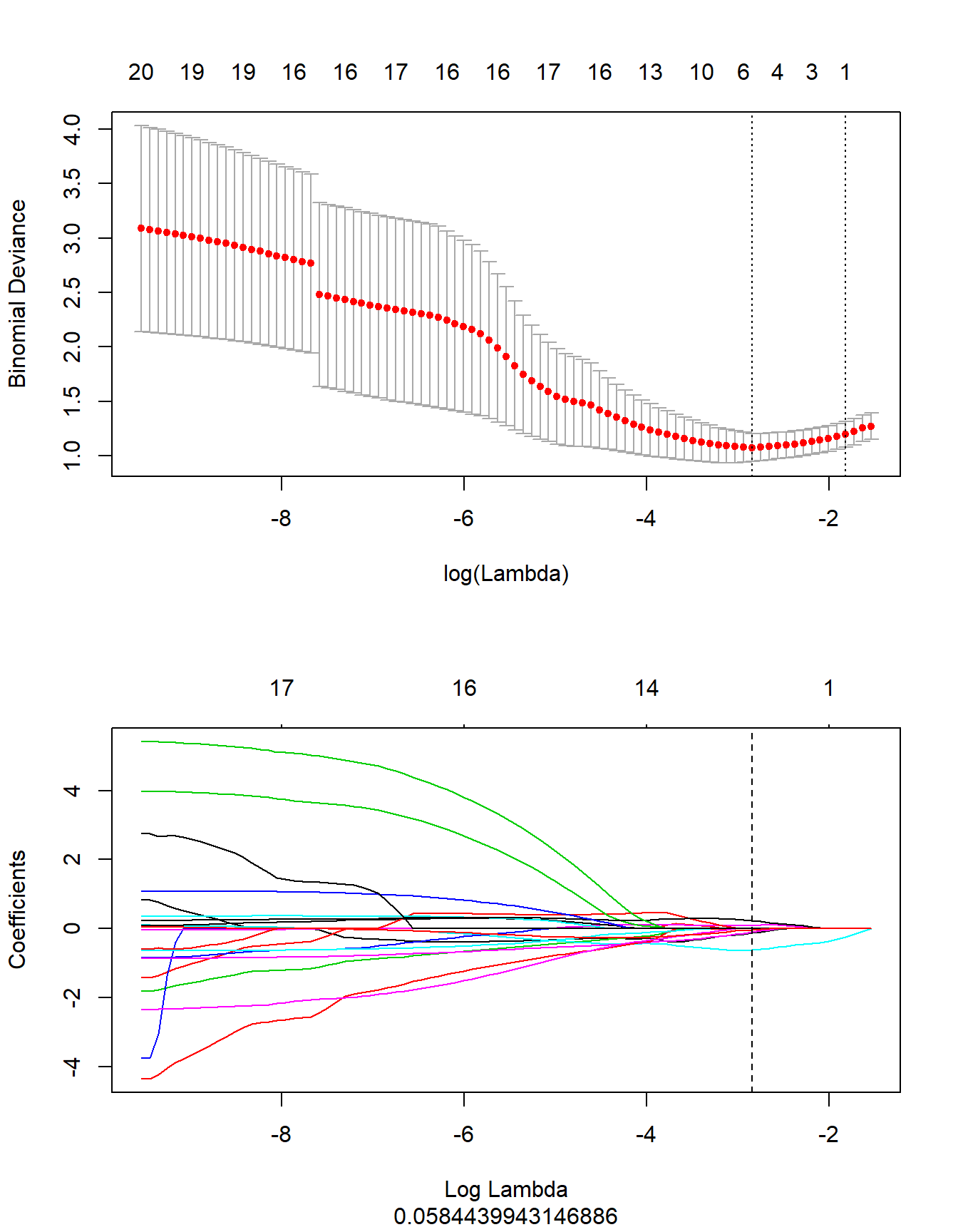

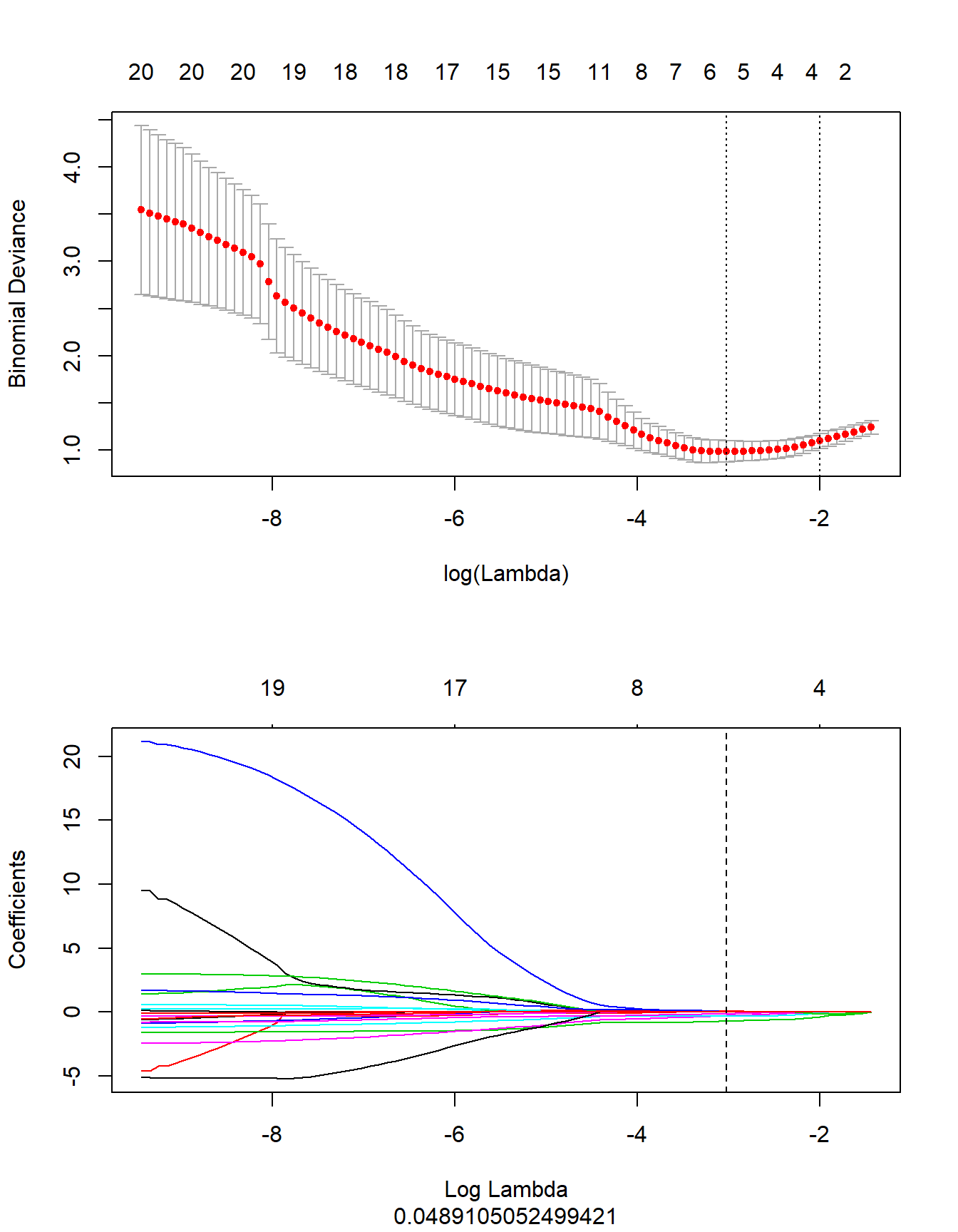

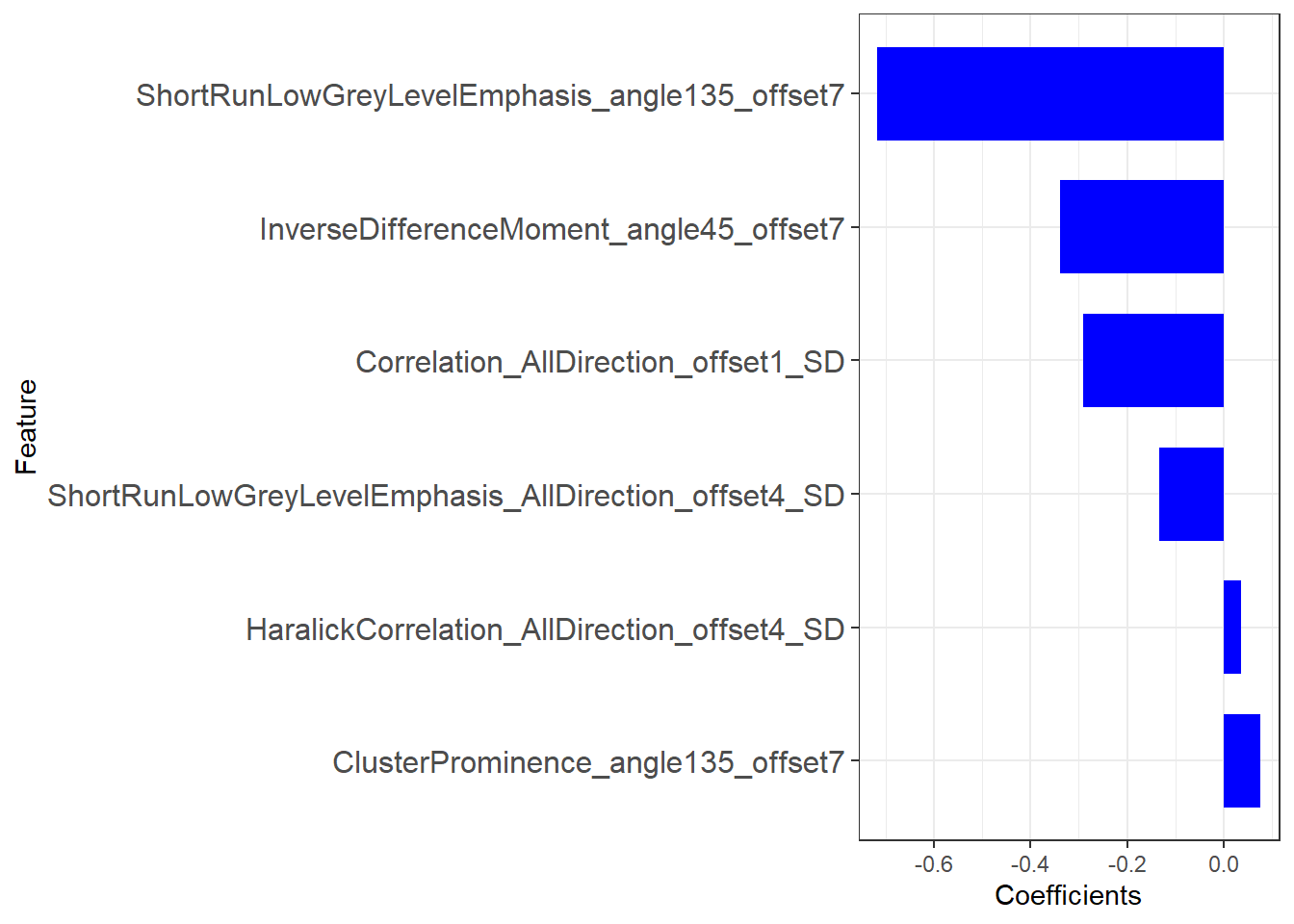

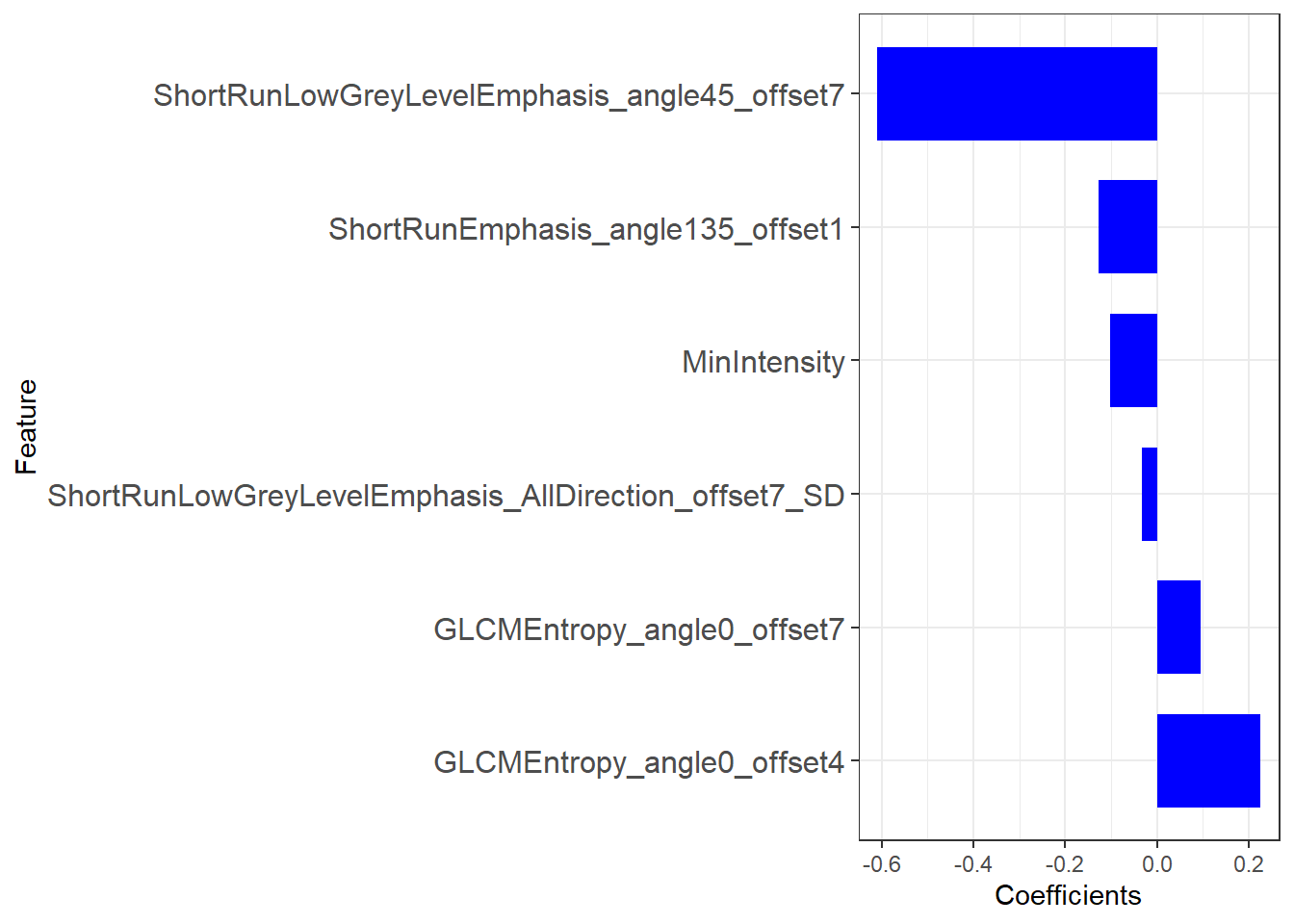

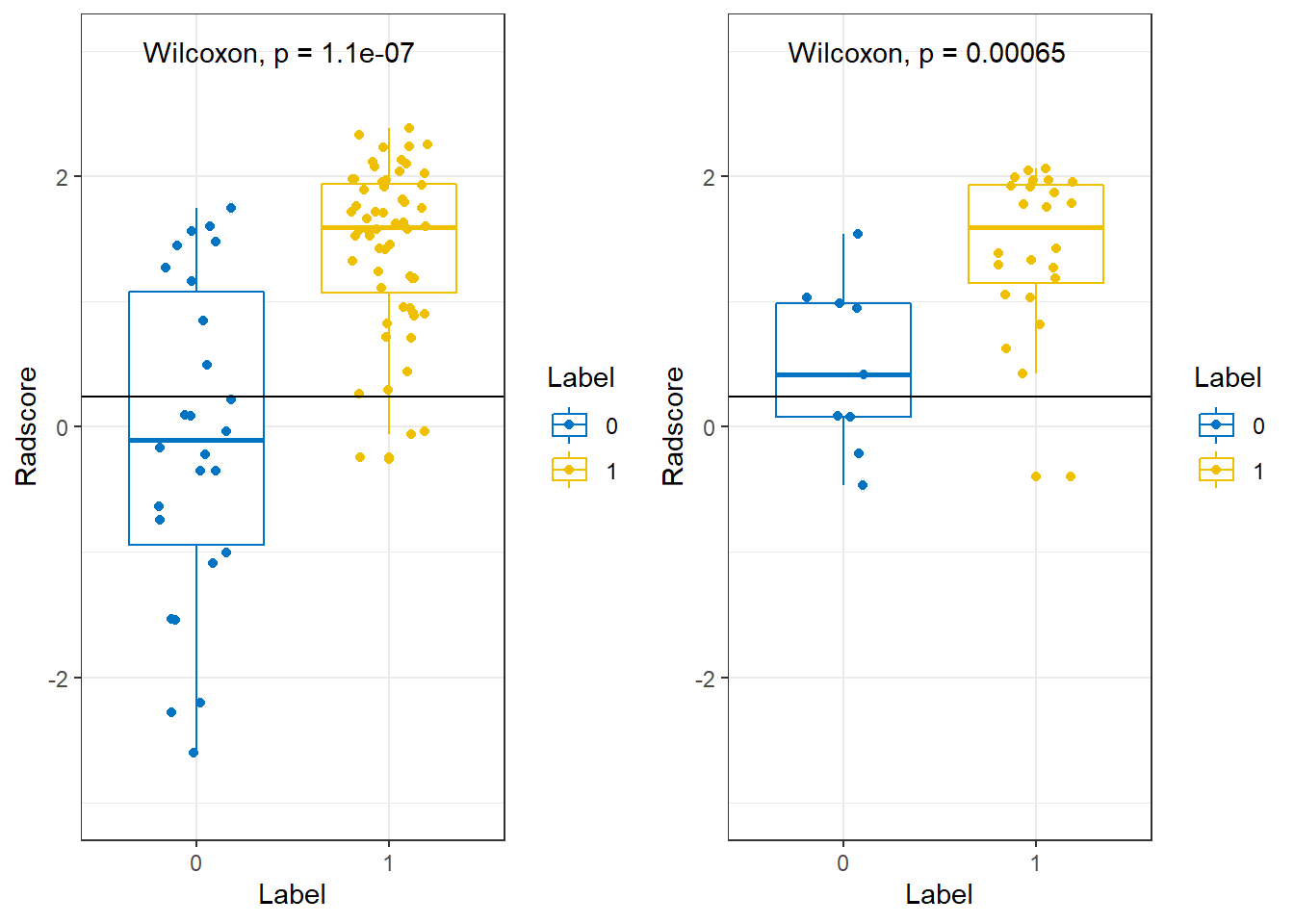

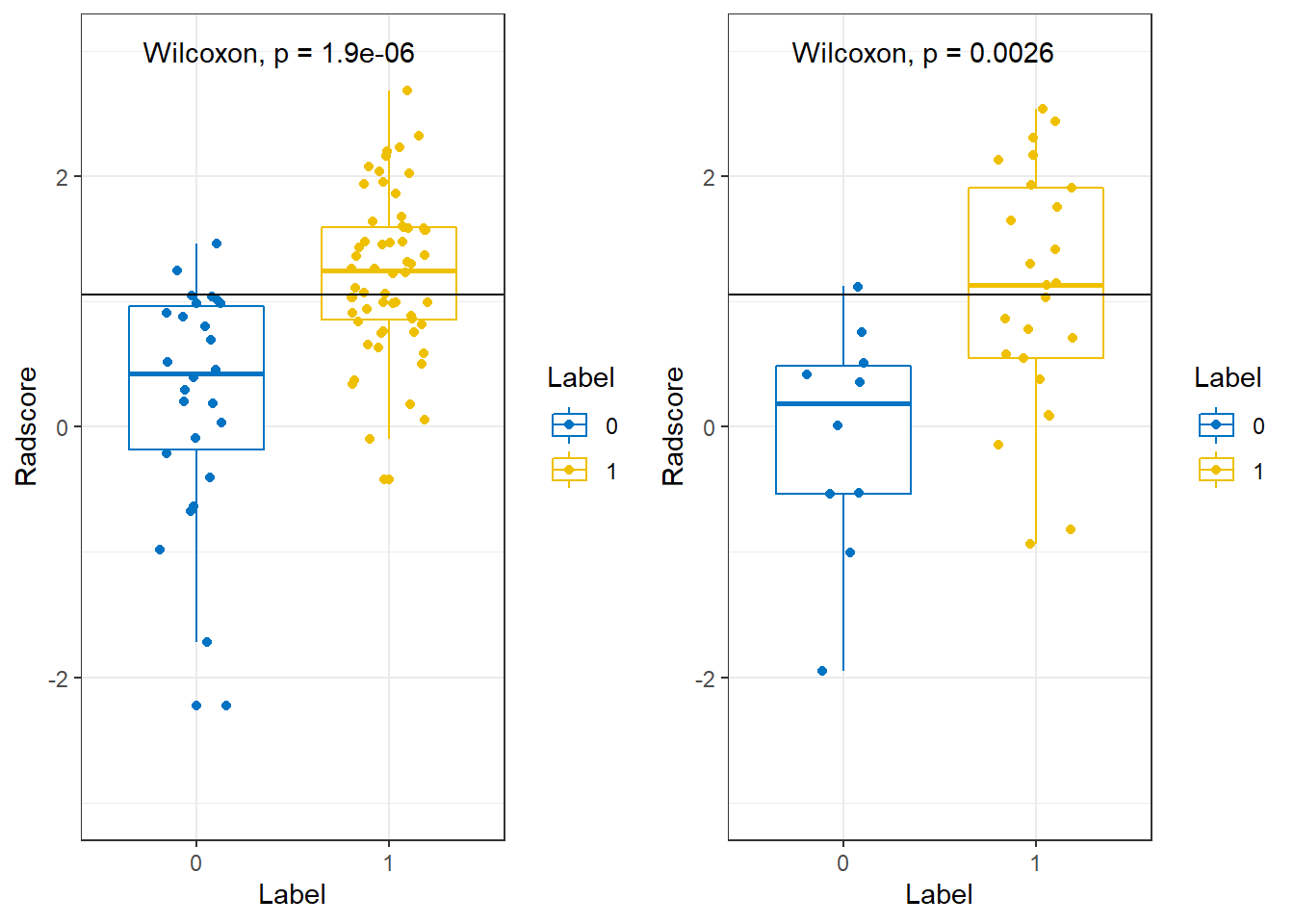


(H)

(F)

(G)

(E)

(D)

(C)

Intranodular rad-score perinodular rad-score

Fig. S1 (A-D) Texture feature selection by using the least absolute shrinkage and selection operator (LASSO) about intronodule, and perinodular rad-score. (E, F) The selected radiomic features and their coefficients about the intronodule, and perinodular rad-score. (G, H) The boxplot shows the intronodule, and perinodular rad-scores between AIS/MIA and IA both in training and validation set.


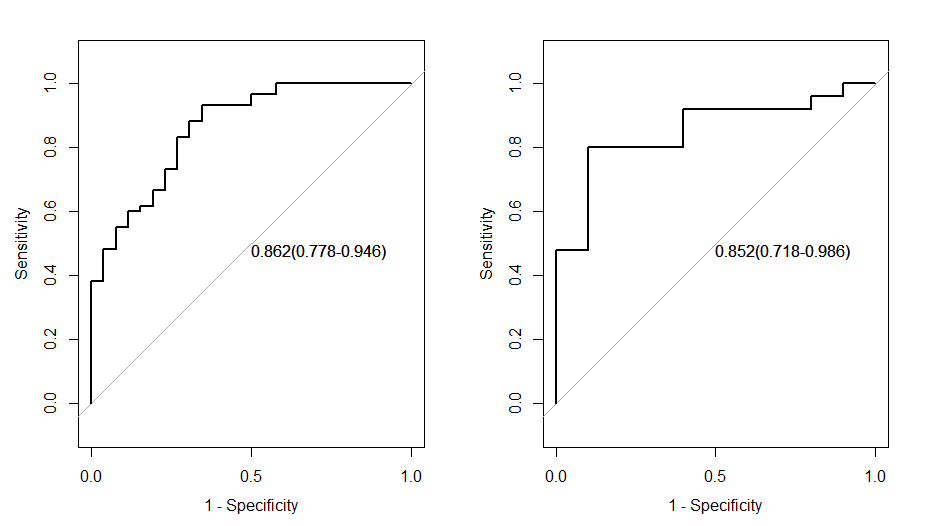


(D)

(C)

(B)

(A)


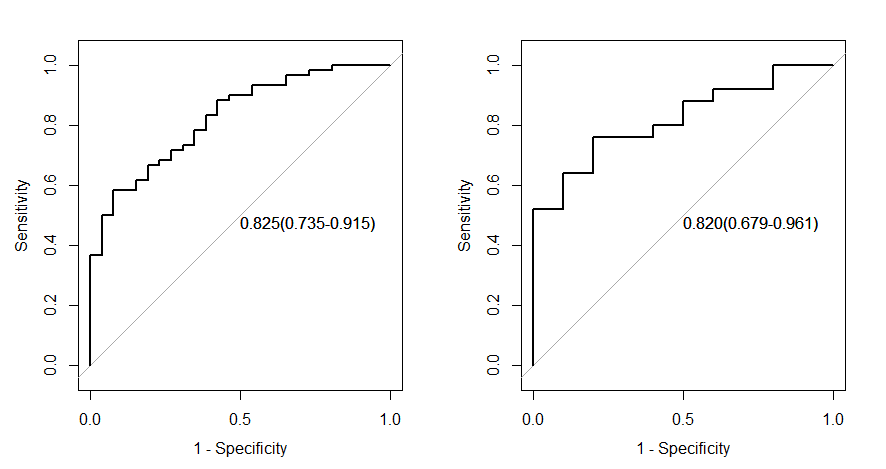


training set validation set

Fig. S2 (A, B) The ROC curve of the intranoduar signatures in the training cohort and the validation cohort, respectively. (C, D) The ROC curve of the perinodular signatures in the training cohort and the validation cohort, respectively.


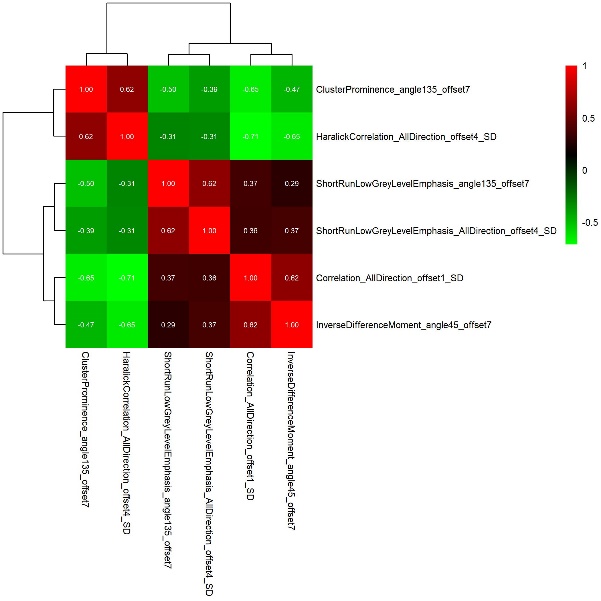

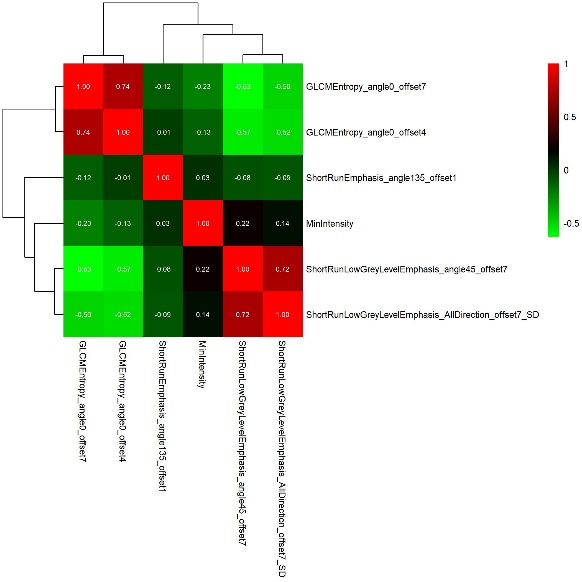


Intranodular heat map perinodular heat map

Fig. S3 the correlation matrix heat map about intranodule and perinodule showing no collinearity between features


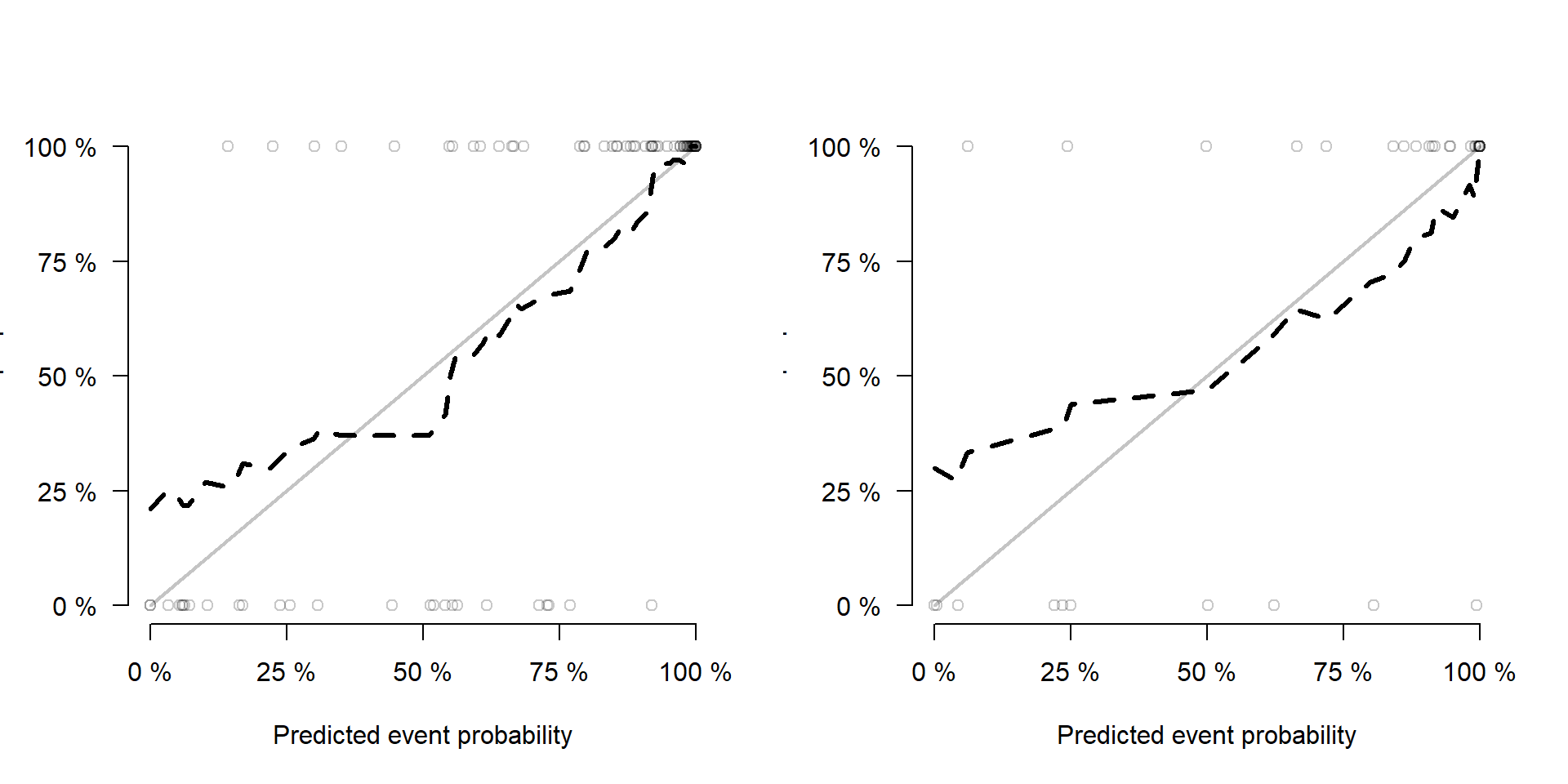


Training set Validation set

Fig. S4 Calibration curve showing the predicted versus actual probability for invasive adenocarcinoma in the training cohort and the validation cohort.

1. **Supplementary Results**

**Calculation formulas for three models and combination clinical-radiomic model**

These features were presented in the rad-score calculated by using the following formula for intranodular, perinodular and gross signatures:

Intranodular rad-score = -0.716*ShortRunLowGreyLevelEmphasis_angle135_offset7+-0.29*Correlation_AllDirection_offset1_SD+0.076*ClusterProminence_angle135_offset7+-0.339*InverseDifferenceMoment_angle45_offset7+-0.134*ShortRunLowGreyLevelEmphasis_AllDirection_offset4_SD+0.036*HaralickCorrelation_AllDirection_offset4_SD + 0.972

Perinodular rad-score = -0.127*ShortRunEmphasis_angle135_offset1+-0.609*ShortRunLowGreyLevelEmphasis_angle45_offset7+0.094*GLCMEntropy_angle0_offset7+-0.103*MinIntensity+0.225*GLCMEntropy_angle0_offset4+-0.032*ShortRunLowGreyLevelEmphasis_AllDirection_offset7_SD + 0.929

Gross radscore = -0.456*ShortRunEmphasis_angle135_offset1External2+0.07*ShortRunEmphasis_angle135_offset1+-0.841*ShortRunLowGreyLevelEmphasis_angle0_offset1+-0.18*LongRunHighGreyLevelEmphasis_AllDirection_offset4_SD+0.378*ClusterProminence_angle135_offset7+-0.47*ShortRunEmphasis_AllDirection_offset1External2+-0.293*LongRunLowGreyLevelEmphasis_angle0_offset4External2+0.544*ShortRunHighGreyLevelEmphasis_AllDirection_offset7_SD + 1.15
